# Clinical practice applicability and relevance to non-specialists of a paediatric EEG online learning tool

**DOI:** 10.1186/s12909-023-05017-2

**Published:** 2024-01-31

**Authors:** Veena Kander, Joanne Hardman, Jo M. Wilmshurst

**Affiliations:** 1 Department of Neurophysiology, Red Cross War Memorial Children’s Hospital, Neuroscience Institute, University of Cape Town, Cape Town, South Africa; 2 Department of Education, University of Cape Town, Cape Town, South Africa; 3 Department of Paediatric Neurology, Red Cross War Memorial Children’s Hospital, Neuroscience Institute, University of Cape Town, 5th Floor ICH, Klipfontein Road, Rondebosch, Cape Town, 7700 South Africa

**Keywords:** Electroencephalography, Paediatric EEG, Online teaching, Low-middle income countries

## Abstract

**Background:**

Paediatric electroencephalography (EEG) training is inadequate amongst healthcare practitioners and technicians managing children with epilepsy in sub-Saharan Africa. An entry level handbook was developed for healthcare practitioners in sub-Saharan Africa and subsequently made globally accessible via the International Child Neurology Teaching Network.

**Aim:**

To investigate the usefulness of a paediatric online EEG handbook.

**Method:**

A survey of the ICNApedia online EEG handbook was circulated (December 2021–June 2022), to all 108 handbook registered participants (39 countries) via the research electronic data capture (REDCap) from the University of Cape Town (UCT).

**Results:**

Fifty participants from 25 countries responded: 8 from high income, 16 upper-middle income, 21 lower-middle income and 5 from low-income. 32 (64%) fully and 18 (36%) partially completed the survey. 35/50 (70%) had completed the handbook and seven respondents had partially completed the handbook. Responses supported the handbook as a good entry point to learn EEGs, especially for paediatrics. Likert scale ratings supported the handbook as relevant for gaining/enhancing knowledge and improving diagnosis and management of patients with confidence. The handbook was considered user friendly, comprehensible, and provided a practical experience. For improving EEG reading skills the handbook helped skills development via reinforcement and good illustrations. 29/32 (90%) of respondents confirmed that they are using learnt skills from the handbook in their current work.

**Conclusion:**

In resource limited settings non-specialist clinicians often provide extended services including EEG interpretation. The survey supports that the handbook is supporting this niche skills area, especially for the accessibility of knowledge gained. The handbook will continue to be adapted in-line with survey feedback.

**Supplementary Information:**

The online version contains supplementary material available at 10.1186/s12909-023-05017-2.

## Key points

• An entry level paediatric EEG handbook was developed for non-specialists.

• Practitioners in real time clinical practice found the handbook to be useful and applicable.

• The handbook was affordable, easy to use and concise.

• The users confirmed change in practice following completion of the handbook.

**Supplementary Information:**

The online version contains supplementary material available at 10.1186/s12909-023-05017-2.

## Introduction

Epilepsy is one of the most common neurological disorders of the central nervous system (CNS) and commonly begins in childhood [1]. There is limited access to quality, reliable and cost-effective health care facilities in resource limited countries (RLCs). Access to diagnostic equipment [EEG and neuroimaging] and personnel is also limited with geographic disparity in distribution (urban vs rural) [2].

Lack of paediatric EEG expertise in Sub-Saharan Africa (SSA) led the researcher to develop a handbook on paediatric EEG interpretation. The handbook aims to improve and increase basic interpretation skills amongst those treating children with epilepsy in SSA for safe practice and not to make them epileptologists. In 2015, the researcher demonstrated that early used of the handbook was effective as a learning tool, although face to face training strengthened the outcome [3].

In 2021, a systematic review was performed on EEG tools for the non-specialist and found that whilst available, they are rarely critiqued for quality of impact [4]. The study found similarities with key concepts taught and that paediatric EEG training was not adequately addressed in some EEG training programs. Of the published studies critiquing existing training programs, the lack of consistency and directness limited comparison [5].

This report aims to assess the usefulness and applicability of the handbook by the users in real-time clinical practice.

## Methodology

We developed a 15-min web-based survey consisting of 26 descriptive and interpretative questions (proforma Supplementary file 1). The survey was emailed to 108 participants from 39 countries who registered to do the online course since it went live in 2016 on the International Child Neurology Association website ICNApedia.org. Subsequently it has been embedded into the epilepsy module of the International Child Neurology Teaching Network (ICNTN) in 2021. Research electronic data capture (REDCap) from the University of Cape Town’s (UCT) web applications were utilised to circulate the survey via the web with weekly eblasts (24th Dec 2021–24th June 2022). In addition, the author sent out emails with REDCap links on a weekly basis. Informed consent was obtained from all the participants. The survey collected data on participants (demographics; hospital location (tertiary, secondary, primary, public, private); whether they treat children and report on paediatric EEGs; training received in paediatric EEG (when it occurred and how long). They were then asked if they completed the handbook or not. The last section focused on their experience and opinion of the handbook. The questions were mainly formatted as drop-down options, where possible with Likert scales, and open-ended questions. Data was analysed and presented using descriptive statistics. Open-ended questions were analysed for themes that emerged in the data rather than imposed deductively [6]. We included all complete surveys and those that answered at least half of the survey questions. The study was approved by the ethics committee of the UCT, Cape Town, South Africa (481/2018).

### Statistical analysis

All the survey data was exported from the online data management software REDCap into Stata v14.2 (StataCorp, College Station, TX, USA) for analysis. Figures presented were generated using Microsoft Excel. Data were presented as proportions since most of the variables collected were categorical and the *p* values were generated using chi square (χ2) or fisher’s exact test. The mean age and the standard deviation of the cohort was reported and to test the association in the different income groups the ANOVA test with a Bonferroni correction was used. The handbook, comparison between the proportions of responders versus non-responders by country income level was assessed using the Z-test. A *p*-value of less than or equal to 0.05 was used to denote statistical significance.

## Results

**Fig. 1:**
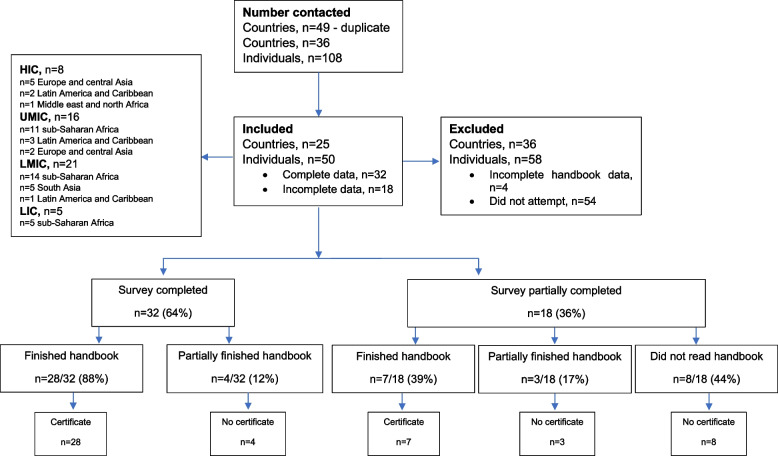
Flow diagram of survey responses

**Fig. 2:**
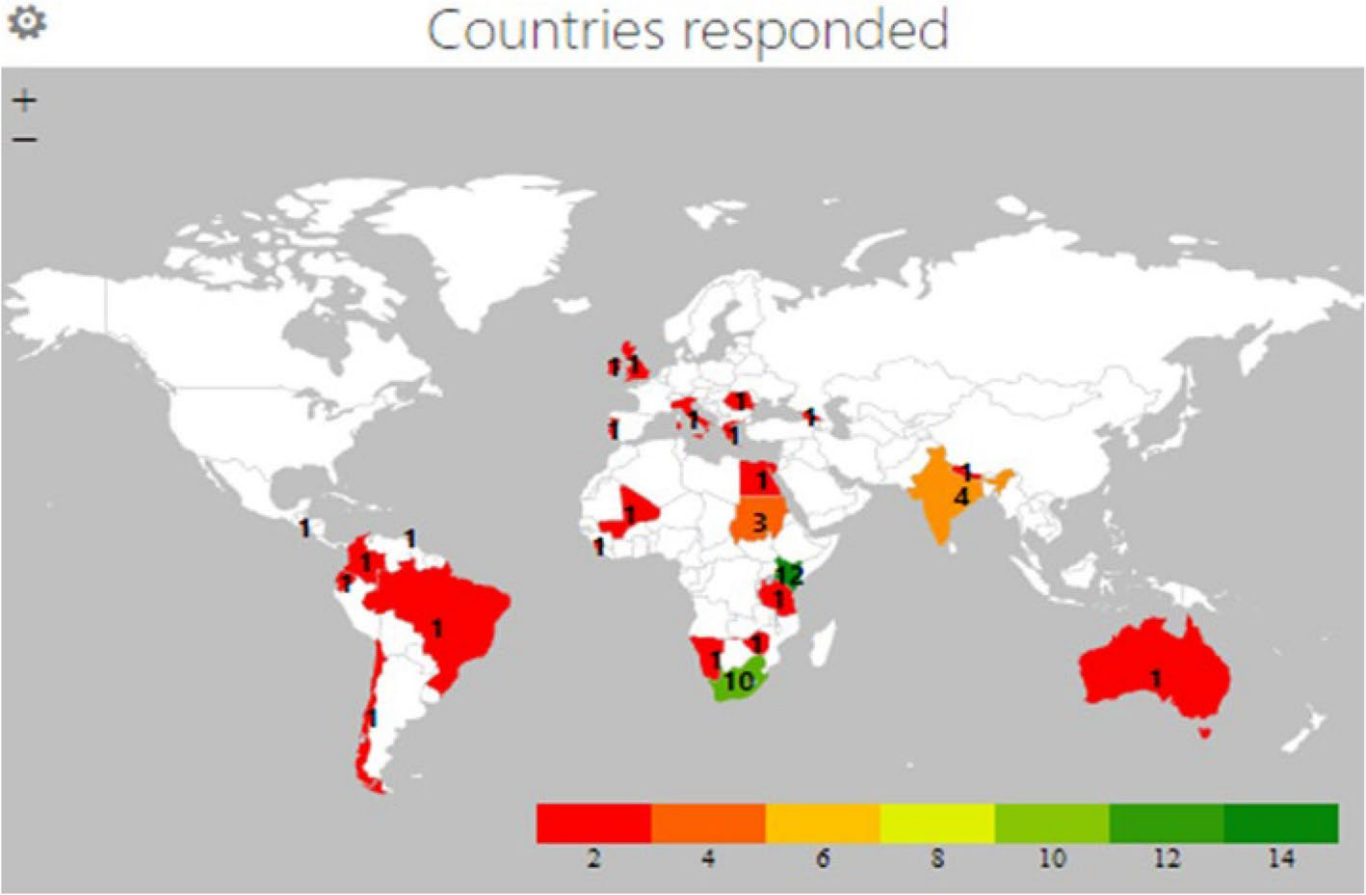
Countries with colour coding for number of responders (red 1–2; orange 3–4; light green 9–10 and medium green 11–12)

We received 50 (46%) out of 108 responses to our survey (Fig. 1). Out of the 50 participants, 35 (70%), obtained a certificate from the online course (Fig. 1). As based on the world bank classification (https://blogs.worldbank.org/opendata/new-world-bank-country-classifications-income-level-2021-2022) the breakdown of the 50 participants and per country is seen in Figs. 1 and 2.

Inclusion of the partially completed survey responses provided valuable additional information from 10 participants who had either completed (7) or partially (3) completed the handbook. 58/108 (54%) did not access the survey, of whom 24 (41%) consented but did not go on to complete the survey. 34 (59%) failed to acknowledge the invitation to be part of the study. The breakdown of the countries that did not respond is available in Supplementary Fig. 1. The comparison of the participants who completed the handbook between the responders and non-responders by income country is seen in Table 1. There is no difference in participants from upper middle-income (UMI) and lower middle-income countries (LMICs), however, there is a threefold number of participants from the high-income countries (HICs) of non-responders compared to the responders. Low-income country (LIC) participants can only be seen in the responders group. 26 (45%) non-respondents who completed the online handbook with certificates failed to complete the survey.

**Table 1:** Comparison of study survey respondents versus non-respondents by income country

**No. of Countries**	**No. of participants**	**Responders-completed HB**	**No. of Countries**	**No. of participants**	**Non-responders-completed HB**	***P*-value**
**HIC (8)**	8	2 (25%)	**HIC (16)**	23	13 (56%)	0.12
**UMIC (7)**	16	11 (69%)	**UMIC (10)**	14	7 (50%)	0.30
**LMIC (7)**	21	18 (86%)	**LMIC (9)**	21	6 (29%)	< 0.001
**LIC (3)**	5	4 (80%)				-

*HB* Handbook, comparison between the proportions of responders versus non-responders by country income level assessed using the Z-test

### Participants information

**Fig. 3:**
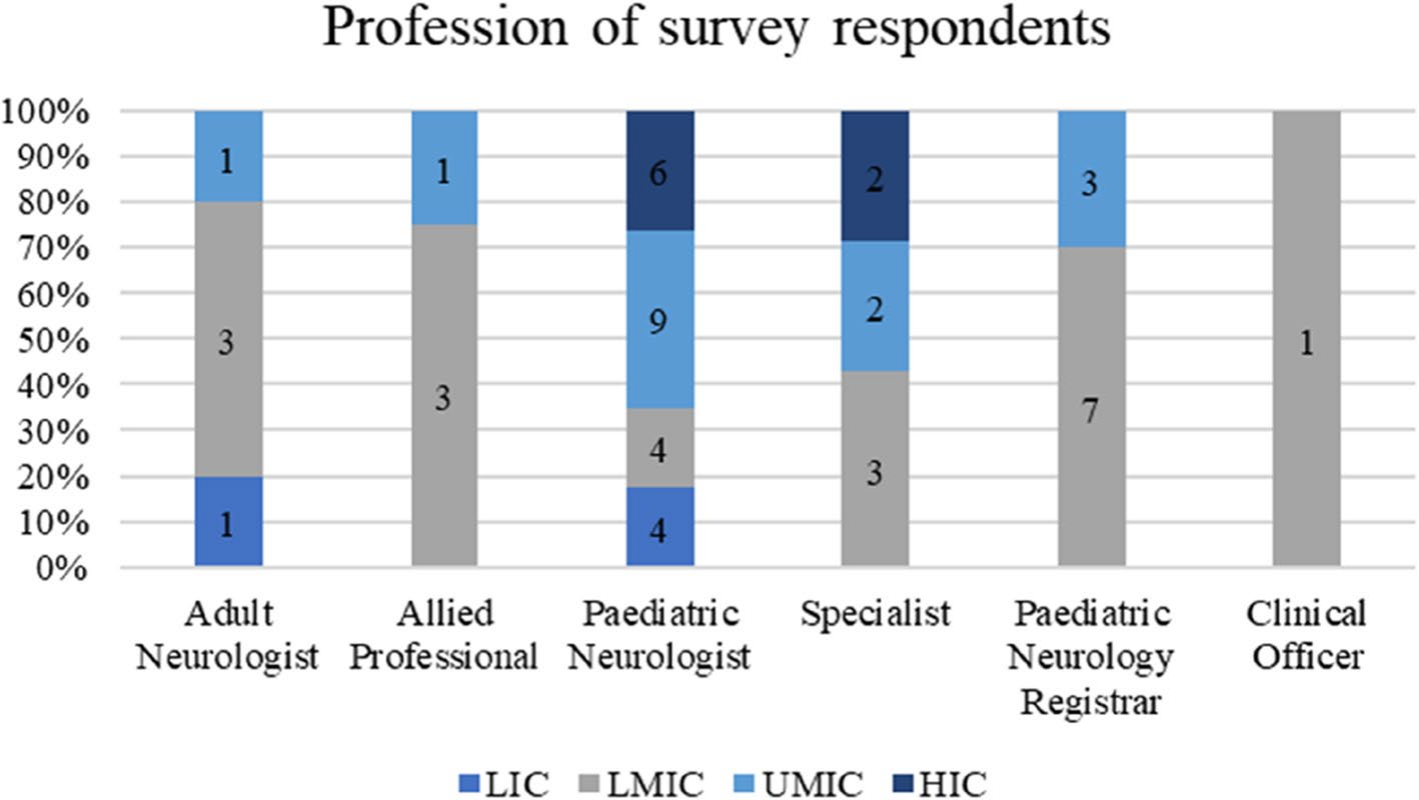
Distribution of the professions by the country income grouping of the survey participants

Overall, there was a female participant preponderance amongst the survey respondents (56%), although most participants from LICs were male (80%). The average age of the study group was 43 years (SD +/-10) with most being paediatric neurologists 23/50 (46%) across the different income countries except for LMICs as illustrated in Fig. 3.

Most participants worked in the tertiary care setting (35) and private (12) and a minority in community care (2) and secondary care (1). 47/50 (94%) are actively involved in the management of children with epilepsy, but only 26 (52%) report paediatric EEGs. 20 (76.9%) reported that they had had previous EEG training. Some participants had multiple exposures of EEG training. The majority,12/20, trained during a fellowship program followed by 11 for online courses. Surprisingly only 3 had training during their registrar rotation who further went on to do fellowship (3) and online courses (2). For those who had undergone EEG training the time ranged from < 3 months to 13 years of training as seen in Table 2. The handbook data is described below in further detail.

**Table 2:** Comparison of respondent characteristics by the country income grouping of the survey participants

**Characteristic**	**Total**	**Low Income Countries**	**Low-Middle Income Countries**	**Upper-Middle Income Countries**	**High Income Countries**	***P*-value**
N	50	5	21	16	8	
Sex of participants						
Female	28 (56.0)	1 (20.0)	11 (52.4)	9 (56.3)	7 (87.5)	0.12
Male	22 (44.0)	4 (80.0)	10 (47.6)	7 (43.8)	1 (12.5)	
Age in years	42.9 (± 10.1)	42.8 (± 6.3)	43.0 (± 11.0)	42.9 (± 11.1)	42.6 (± 9.5)	0.63
Profession						
Adult Neurologist	5 (10.0)	1 (20.0)	3 (14.3)	1 (6.3)	0	0.11
Allied Professional	4 (8.0)	0	3 (14.3)	1 (6.3)	0	
Paediatric Neurologist	23 (46.0)	4 (80.0)	4 (19.1)	9 (56.3)	6 (75.0)	
Specialist	7 (14.0)	0	3 (14.3)	2 (12.5)	2 (25.0)	
Other	11 (22.0)	0	8 (38.1)	3 (18.8)	0	
Specialisation						
Developmental paediatrician	2/7 (28.6)	-	0	1/2 (50.0)	1/2 (50.0)	0.20
Neonatology	1/7 (14.3)	-	0	0	1/2 (50.0)	
Paediatrician	4/7 (57.1)	-	3/3 (100)	1/2 (50.0)	0	
Allied professional						
Neurophysiologist	4/4 (100)	-	3/3 (100)	1/1 (100)	-	1.00
Other						
Clinical officer	1/11 (9.1)	-	1/8 (12.5)	0	-	1.00
Paediatric neurology registrar	10/11 (90.9)	-	7/8 (87.5	3/3 (100)	-	
Hospital						
Community clinic	2 (4.0)	0	0	1 (6.3)	1 (12.5)	0.31
Private	12 (24.0)	0	6 (28.6)	5 (31.3)	1 (12.5)	
Secondary care	1 (2.0)	0	0	0	1 (12.5)	
Tertiary care	35 (70.0)	5 (100)	15 (71.4)	10 (62.5)	5 (62.5)	
Treat paediatric epilepsy	47 (94.0)	5 (100)	19 (90.5)	16 (100)	7 (87.5)	0.56
Report Paediatric EEG	26 (52.0)	3 (60.0)	12 (57.1)	9 (56.3)	2 (25.0)	0.46
Previous EEG training	20/26 (76.9)	2/3 (66.7)	10/12 (83.3)	6/9 (66.7)	2/2 (100)	0.74
EEG training						
Registrar	3 (6.0)	0	2 (9.5)	1 (6.3)	0	1.00
Fellowship program	12 (24.0)	2 (40.0)	5 (23.8)	3 (18.8)	2 (25.0)	0.83
Online course	11 (22.0)	1 (20.0)	7 (33.3)	3 (18.8)	0	0.30
Other	2 (4.0)	0	2 (9.5)	0	0	-
Hospital EEG department	1 (2.0)	0	1 (4.8)	0	0	-
Private clinic	1 (2.0)	0	1 (4.8)	0	0	-
Training time						
< 3 months	1/20 (5.0)	0	1/10 (10.0)	0	0	0.88
3–6 months	6/20 (30.0)	1/2 (50.0)	4/10 (40.0)	1/6 (16.7)	0	
7–12 months	10/20 (50.0)	1/2 (50.0)	4/10 (40.0)	3/6 (50.0)	2/2 (100)	
Other	3/20 (15.0)	0	1/10 (10.0)	2/6 (33.3)	0	
Completion of online course						
Yes	35 (70.0)	4 (80.0)	18 (85.7)	11 (68.8)	2 (25.0)	***0.02***
Partially	7 (14.0)	0	1 (4.8)	4 (25.0)	2 (25.0)	
No	8 (16.0)	1 (20.0)	2 (9.5)	1 (6.3)	4 (50.0)	
Reason for non-completion						
Didn’t have time to complete	6/11 (54.6)	1/1 (100)	0	3/3 (100)	2/4 (50.0)	0.49
Not aware of the online course	1/11 (9.1)	0	1/3 (33.3)	0	0	
Didn’t read it	1/11 (9.1)	0	1/3 (33.3)	0	0	
Didn’t receive the handbook	2/11 (18.2)	0	1/3 (33.3)	0	1/4 (25.0)	
I don’t have it	1/11 (9.1)	0	0	0	1/4 (25.0)	
Year of completion						
2017	7/34 (20.6)	1/4 (25.0)	3/17 (17.7)	2/11 (18.2)	1/2 (50.0)	0.97
2018	1/34 (2.9)	0	1/17 (5.9)	0	0	
2019	3/34 (8.8)	0	1/17 (5.9)	1/11 (9.1)	1/2 (50.0)	
2020	3/34 (8.8)	0	2/17 (11.8)	1/11 (9.1)	0	
2021	13/34 (38.2)	2/4 (50.0)	6/17 (35.3)	5/11 (45.5)	0	
2022	7/34 (20.6)	1/4 (25.0)	4/17 (23.5)	2/11 (18.2)	0	
Completion time						
< 1 week	8/35 (22.9)	1/4 (25.0)	3/18 (16.7)	4/11 (36.4)	0	0.94
1–2 weeks	3/35 (8.6)	0	3/18 (16.7)	0	0	
3–4 weeks	12/35 (34.3)	2/4 (50.0)	6/18 (33.3)	3/11 (27.3)	1/2 (50.0)	
> 1 month	1/35 (2.9)	0	1/18 (5.6)	0	0	
Other	11/35 (31.4)	1/4 (25.0)	5/18 (27.8)	4/11 (36.4)	1/2 (50.0)	
Completion time (Other)						
5 weeks	1/11 (9.1)	0	1/5 (20.0)	0	0	
6 weeks	1/11 (9.1)	0	0	0	1/1 (100)	
2 months	2/11 (18.2)	0	1/5 (20.0)	1/4 (25.0)	0	
10 weeks	1/11 (9.1)	0	1/5 (20.0)	0	0	
3 months	1/11 (9.1)	0	0	1/4 (25.0)	0	
4 months	2/11 (18.2)	0	1/5 (20.0)	1/4 (25.0)	0	
6 months	2/11 (18.2)	1/1 (100)	0	1/4 (25.0)	0	
8 months	1/11 (9.1)	0	1/5 (20.0)	0	0	
Reason for the delay - Busy schedule	11/11 (100)	1/1 (100)	5/5 (100)	4/4 (100)	1/1 (100)	-
Online facilities	20/39 (52.6)	2/4 (50.0)	12/18 (66.7)	5/13 (38.5)	1/4 (25.0)	0.42
Reasons for not using the chat						
Did not need it	6/19 (31.6)	0	3/6 (50.0)	2/8 (25.0)	1/3 (33.3)	0.89
Did not understand how to use it	2/19 (10.5)	0	1/6 (16.7)	1/8 (12.5)	0	
Was not aware of availability of chat	11/19 (57.9)	2/2 (100)	2/6 (33.3)	5/8 (62.5)	2/3 (66.7)	

*P*-value of ≤ 0.05 was considered statistically significant

Proportions (%) for the columns reported as n/N (%) (except if data is missing - denominator added in the n column)

Continuous data expressed as mean (± standard deviation (SD)) since data is normally distributed

*P*-values derived usingChi-squared test/ Fishers exact test for categorical data

*P*-values derived using Anova test for variance with a Bonferroni correction for continuous data

### Online handbook course

Close to three quarters of the respondent 35/50 (70%) completed the online course. Respondents were balanced from the LIC, LMIC and UMICs. But this differed for HICs where half of the respondents who had registered did not attempt the online course is available in Supplementary Fig. 2. Seven partially completed the online course and eight did not. As such they read some of the chapters and either attempted the post chapter quizzes or not. The consensus for this was “not having time to complete the handbook” in (6/11). The year of completion ranged across 6 years (2017–2022). Completion time was variable from less than a week to 8 months owing to having a “busy schedule” in 11 as reason for delay. 11/19 (58%) were not aware they could use the chat to clarify any information.

### Experience and opinion of the handbook – Likert scale

**Fig. 4:**
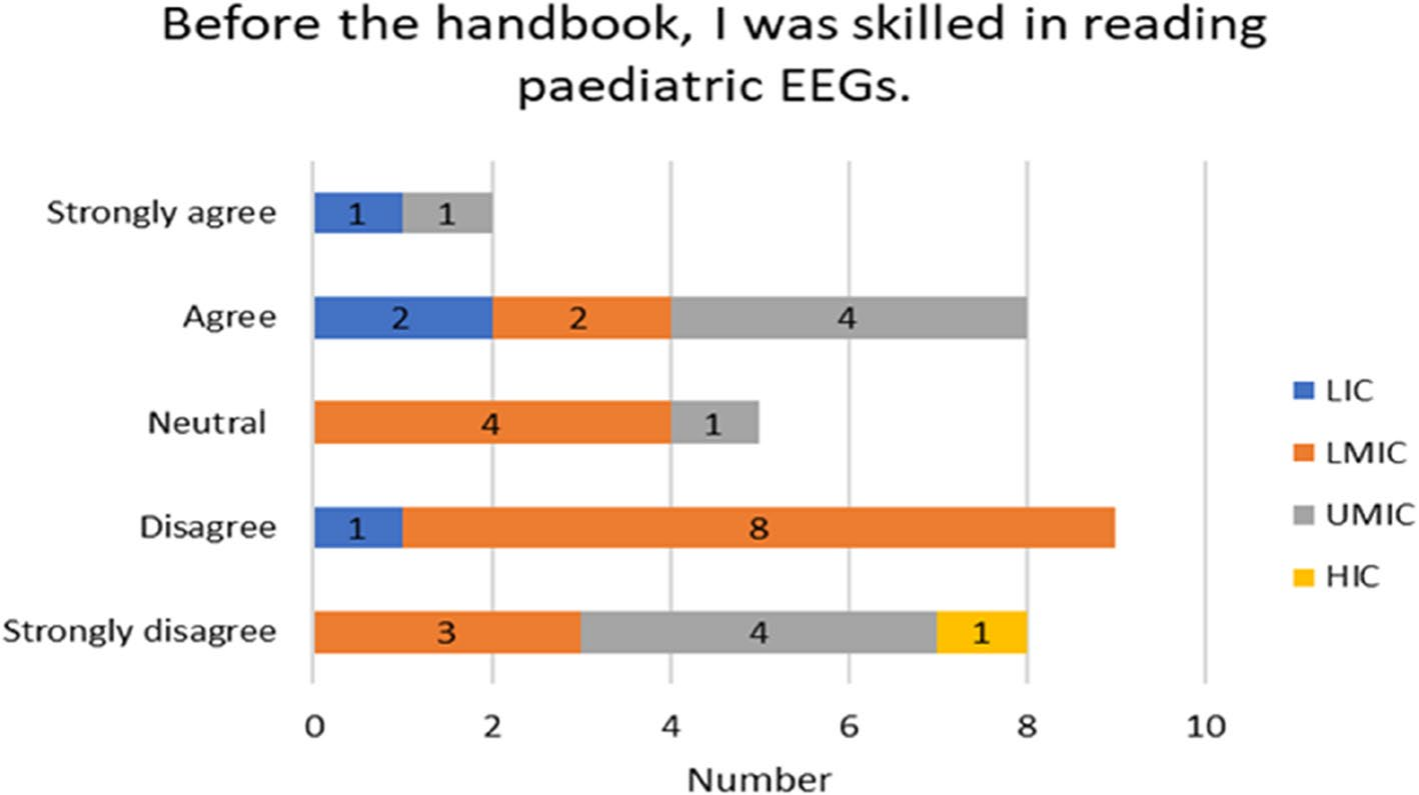
Prior handbook skills in paediatric interpretation *n* = 32

**Fig. 5:**
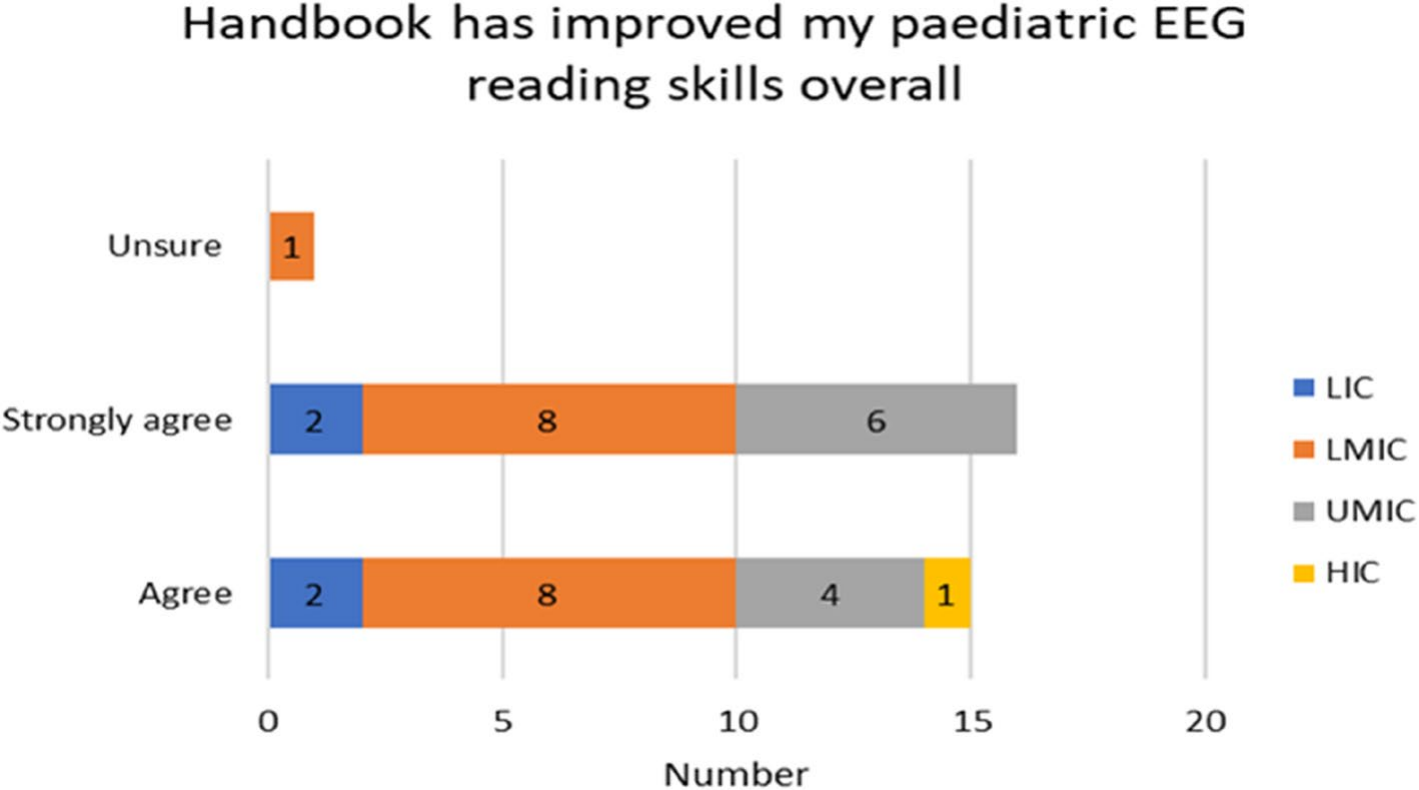
Reading skills *n* = 32

**Fig. 6:**
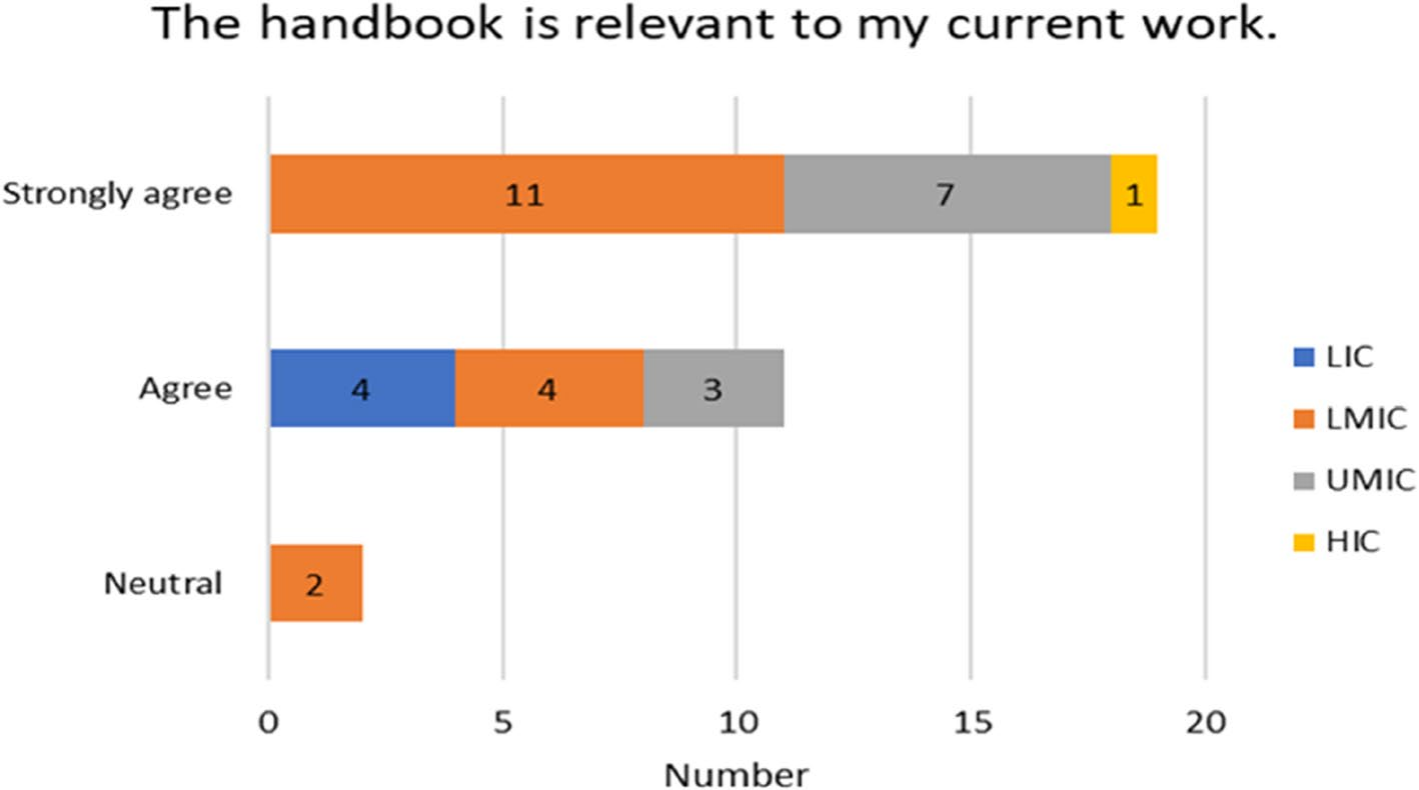
Relevance of handbook to current work *n* = 32

**Fig. 7:**
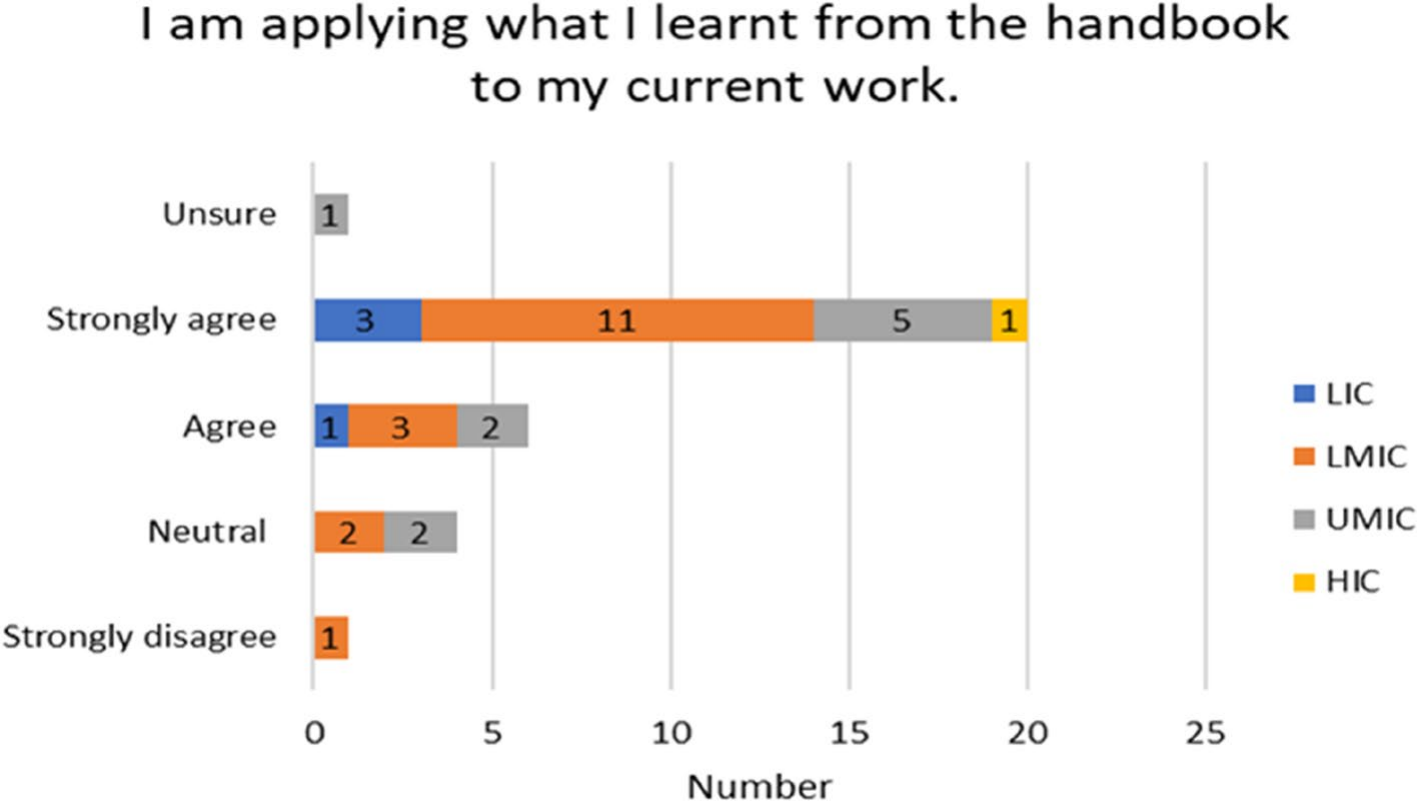
Application of handbook to current work *n* = 32

**Fig. 8:**
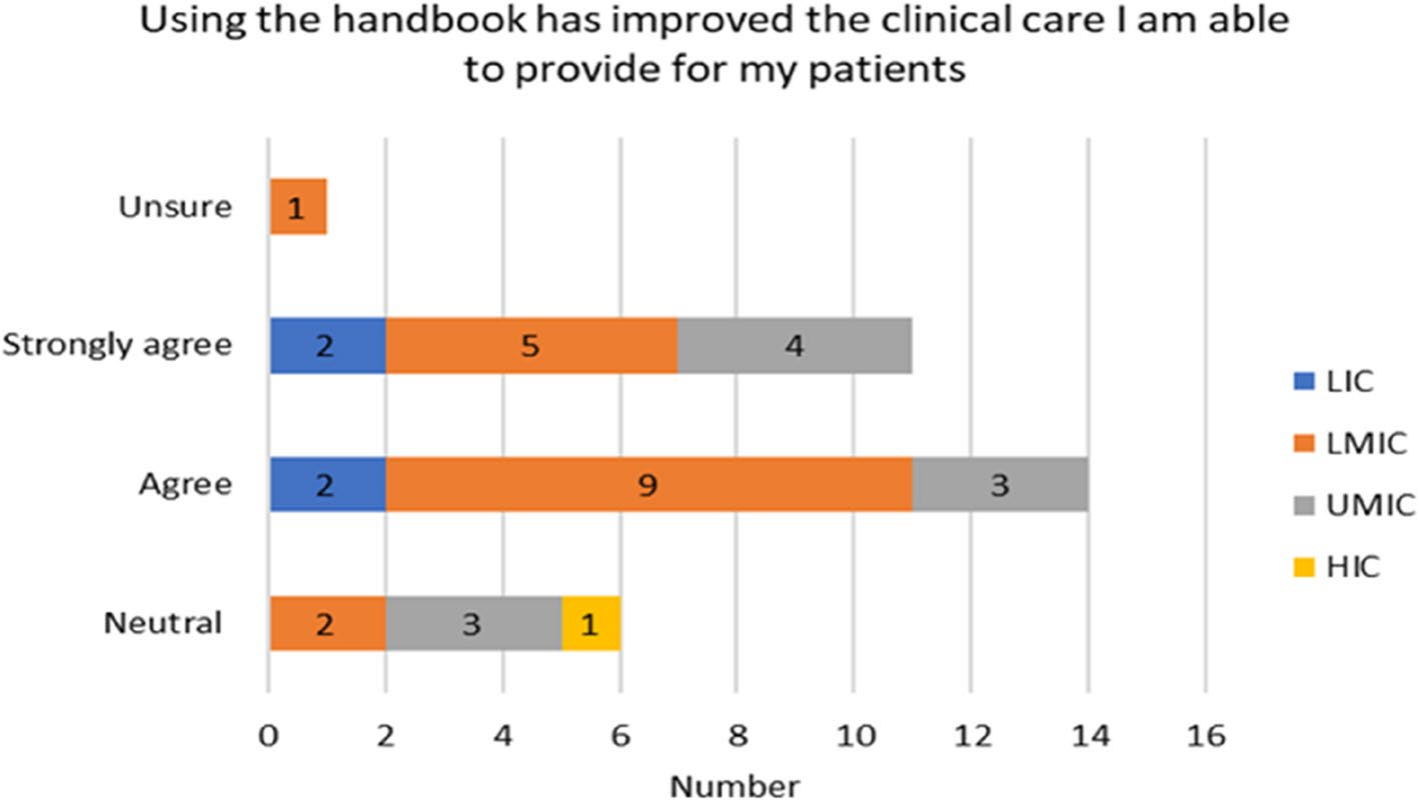
Improved clinical care *n* = 32

The experiences of the respondents with respect to already having skills in reading paediatric EEGs varied by income country. Over half of the participants 17/32 (53%) on the Likert scale disagreed/strongly or disagreed with not having paediatric skills before the handbook. A higher proportion of responses came from LMIC (11) seen in Fig. 4. 30/50 (60%) found the handbook relevant to their current work and (2) stayed neutral (Fig. 6). For the rest of the experiences and opinions Figs. 5, 6, 7 and 8, the respondents were generally agreeing/strongly or agreeing. 30 (60%) gave additional explanatory comments for their rating. Out of which 2 failed to complete all explanatory comments although they both strongly agreed/agreed on their experience and opinion of the handbook. The authors both analysed the qualitative data for themes and agreement between the authors on the identification of themes was obtained. Thematic analysis using descriptive themes was done. The following themes emerged in relation to each question. These are provided in the analysis below and discussed later in the text.

Thirty participants responded to the question whether using the handbook has improved their paediatric EEG reading skills overall (Fig. 5) and gave reasons for their rating. Three sub-themes emerged inductively from the data.

#### Theme 1: improvement of paediatric reading skills

**Table:** 

**1) User friendly**
Participant 25: Simplicity in explanation.
Participant 26: Easy.
Participant 28: The knowledge and skills in the book were useful.
**2) Comprehensible (helped my knowledge)**
Participant 6: Reaffirmed knowledge.
Participant 3: I was able to understand the EEG rhythms and identify them.
Participant 14: I learnt the basic EEG knowledge and interpretation skills.
**3) Practical examples**
Participant 21: Used as a refresher.
Participant 18: I got more comfortable with paediatric EEGs and my confidence grew.

When asked whether using the handbook has improved paediatric EEG reading skills in the following topics is available in Supplementary Fig. 3.1–3.4. Here again 30 participants gave their reasons for their rating. The following two sub-themes emerged here: -

#### Theme 2: identification of basic concepts in EEG reading

**Table:** 

**1) Reinforcement**
Participant 10: Repetition of concepts.
Participants 28: The chapter on identification of EEG abnormalities was useful.
**2) Good illustrations**
Participant 7: The book was well illustrated.
Participant 19: The sections were presented in a clear manner.

Twenty-nine participants are using learnt skills from the handbook in their current work (Fig. 7). Only one participant strongly disagreed “I see adult patients only, but the course did help me in getting the basic concepts of EEG. I am still trying to get my hospital to purchase an EEG machine”. The following theme stood out from the responses.

#### Theme 3: relevance - to application

**Table:** 

Participant 18: I am a neurophysiologist working with EEGs every day.
Participant 20: I am currently a neurology trainee hence the skills learnt are quite relevant.
Participant 19: I daily encounter patient with epilepsy and EEG knowledge is key.

In relation to the handbook’s impact on the improvement of respondents’ clinical care (Fig. 8) elicited one central theme. This is illustrated with quotes drawn from the data below.

#### Theme 4: improved reading of EEG

**Table:** 

Participant 18: I can confidently give back feedback on EEGs and provide more sufficient Health care to my patients.
Participant 20: The skills are useful in formulating diagnosis and choosing appropriate intervention for the patients.
Participant 22: It became evident in my practice.
Participant 31: My knowledge for epilepsy has improved a lot and how I manage the patients based on the EEG abnormalities I can identify after the course.

When asked whether they would recommend this handbook to others is available in Supplementary Fig. 4, three sub-themes emerged in the data and are reported below.

#### Theme 5: recommendation of handbook

**Table:** 

**1) Develops understanding**
Participant 16: Well researched book.
Participant 17: Best introduction of EEGs that I have found.
**2) User friendly**
Participant 12: The handbook does explain the basic concepts in a simplified manner.
Participant 7: Easy and concise.
**3) Affordability**
Participant 9: Although a basic course, it is affordable and accessible.

In response to suggestions to improve the course, two sub-themes emerged below. We can see that the addition of videos and case studies to the handbook would improve the overall course according to these respondents.

#### Theme 6: improvement to course

**Table:** 

**1) Videos**
Participant 12: Some online video lectures to clarify difficult concepts would be helpful.
Participant 22: Online video sessions.
**2) Case studies**
Participant 5: A few case studies would be helpful.
Participant 20: Include patient or clinical scenarios as examples.

The impact of Coronavirus disease (COVID) lockdown ensured that much interaction over the past two years has been online. With this in mind, respondents were asked whether the interface that they are presented with could be improved. One theme emerged: Interaction. The need for interaction in online spaces is a recurrent issue in online learning/teaching platforms and is illustrated in the following quotes [7].

#### Theme 7: interaction

**Table:** 

Participant 5: Interaction with others may be useful, having a weekly online meeting may also be useful.
Participant 6: I would suggest more inactive activities.

Finally, the participants were asked to list any positive aspects of taking the course. Twenty-nine participants indicated that one of the most significant achievements of the handbook lies in its accessibility as is illustrated below.

#### Theme 8: accessibility for knowledge

**Table:** 

Participant 9: Short, accessible. A good introduction to EEGs.
Participant 7: It makes you clear and indications of use of EEG.
Participant 11: It provides foundational knowledge and understanding.
Participant 18: Gain more knowledge on child EEG.

## Discussion

The questions this study sought to answer were whether the online handbook is useful, whether the participants liked the handbook and their reasons for this. This entry level handbook was originally developed for healthcare practitioners in sub-Saharan Africa and has since been added to the epilepsy module of the International Child Neurology Teaching Network (ICNTN) making it accessible across the world covering high to low-income countries. The handbook as stated promotes entry level safe practice for the use of EEG as a tool.

30/50 (60%) found the handbook relevant to their work, so it was not surprising that the majority of the participants (33) working with children with epilepsy responded. In our study, our findings favoured a female preponderance [8]. Previous studies support that females are more likely to complete online courses whilst there are mixed gender results at partaking in surveys [8].

When comparing data from Table 1, the proportion of respondents who completed the handbook from the LMIC was significantly higher than the non-respondents who completed the handbook (*p* < 0.001). LIC’s were only present among the responders.

Six out of the eleven participants that partially or did not complete the handbook with the agreement of “not having time to complete”, this is a common clinical and paediatric theme that needs resources that are “digestible”. The above themes, user friendly, accessible, and comprehensive fulfils that resource.

More participants from LMIC (11) and UMIC (4) disagreed respectively with the notion that they were skilled in reading the EEG before the handbook compared to those from LIC (3) or UMIC (5) who strongly agreed.

For the rest of the questions on the handbook, participants’ experiences, and opinions were positive with an overall agree/strongly or agree across all income countries.

The qualitative data was analysed by VK and JH and at times a single or several sub-themes emerged. The themes documented will be used for the ongoing upscaling of the handbook. The theme that stood out most was the accessibility of knowledge gained. Other sub-themes arose such as having more case studies with supporting EEG waveforms; availability of online sessions for interaction to discuss information and assist with clarification, as we all experienced only online interface during the COVID pandemic.

There is a huge need for more training in neurophysiology. Further of the existing published studies critiquing existing training programs the lack of consistency and directness limited comparison [5].

### Limitations

This is a qualitative subjective study and whilst doctors claimed to have learned from this tool this does not necessarily guarantee that they have genuinely acquired knowledge. The main study limitation related to participants failing to complete the survey even after they received weekly email eblasts for 6 months including personal emails with the survey URL. Almost half failed to complete the survey 58/108 (54%) even though 24 (41%) consented to participate in the survey. 26 (45%) of the participants completed the handbook with certificates but failed to complete the survey. The non-respondents could have added valuable information. Further statistical reports for “medians” could not be performed owing to the data set up being categorical responses. Pre and post testing was not performed in this cohort but has been undertaken in a previous group.

## Conclusion

Participants found the handbook to be useful both for their clinical practice and for the accessibility of the knowledge contained in the book. The study was aimed at participants from resource limited settings. The handbook is an entry point to equip clinicians for safe practice to enable them to also undertake more complex training if needed. The handbook is central to EEG training and of especial relevance to the African Paediatric Fellowship Program (APFP) which trains paediatric neurologists from sub-Saharan Africa [9]. These qualified paediatric neurologists return to their countries and at times are the only paediatric neurologist in the country. They recruit a colleague to be trained in assisting them with performing EEGs. This handbook is also used in the curriculum to train EEG technicians as well as clinicians.

### Supplementary Information

**Additional file 1.** PES Handbook Questionnaire.

**Additional file 2: Supplementary Figure 1.** Countries with colour coding for number of non-responders (red 1–2; orange 3–4; and dark green 11–12).

**Additional file 3: Supplementary Figure 2.** Completion of online course by the country income grouping of the survey participants.

**Additional file 4: Supplementary Figure 3.1.** Identification of normal waveforms *n* = 32. **Supplementary Figure 3.2.** Identification of artifacts *n* = 32. **Supplementary Figure 3.3.** Identification of abnormalities *n* =32. **Supplementary Figure 3.4.** Identification of activation procedures *n* = 32.

**Additional file 5: Supplementary Figure 4.** Recommendation of handbook and training program *n* = 32.

## Data Availability

The datasets used and/or analysed during the current study available from the corresponding author on reasonable request.

## Abbreviations

CNS: Central nervous system
COVID: Coronavirus disease
EEG: Electroencephalography
HIC: High-income country
ICNTN: International Child Neurology Teaching Network
JH: Joanne Hardman
LIC: Low-income country
LMIC: Lower middle-income country
REDCap: Research electronic data capture
RLCs: Resource limited countries
SSA: Sub-Saharan Africa
UCT: University of Cape Town
UMIC: Upper middle-income country
URL: Uniform Resource Locators
VK: Veena Kander

## Declarations

This article is based on the research I conducted for my Doctor of Philosophy Degree in Neurophysiology. In relation to the journal’s position on issues involved in ethical publication: We confirm that we have read the Journal’s position on issues involved in ethical publication and affirm that this report is consistent with those guidelines.
