# Supplementary material for: Clinical practice applicability and relevance to non-specialists of a paediatric EEG online learning tool
Source: BMC Med Educ. 2024 Jan 31;24:102. doi: 10.1186/s12909-023-05017-2 (PMC10829391; doi:10.1186/s12909-023-05017-2)
Supplement: Supplementary file 1 — Additional file 1. PES Handbook Questionnaire. [file 12909_2023_5017_MOESM1_ESM.pdf]

PES Handbook Questionnaire

Dear Sir/Madam,

As part of my PhD programme, I am undertaking a survey study to develop an apprenticeship training program for healthcare professionals from sub Saharan Africa in basic paediatric electroencephalography skills.

The aim of the study is to understand the impact, change in knowledge and practice using an online paediatric course and whether chat/online support is important?

To this end, I kindly request that you complete the following short handbook questionnaire.

Thank you!

Participant information

Please choose your gender	◯ male<br>◯ female<br>◯ other<br>(Please choose your gender)
Please fill in your age	__________________<br>(Please only fill in your age in number format)
Please fill in your profession	◯ Adult neurologist<br>◯ Paediatric neurologist<br>◯ Medical officer<br>◯ Specialist<br>◯ Allied Professional<br>◯ Other<br>(Please choose your profession)
Please fill in your speciality field, if relevant.	__________________
Please fill in your allied profession field, if relevant.	__________________
If OTHER, please specify your profession.	__________________
Please fill in your primary country of residence.	__________________<br>(Please only fill in your country)
What type of hospital or clinical setting do you usually work in?	◯ Tertiary care (healthcare specialist in a large hospital)<br>◯ Secondary care<br>◯ Private<br>◯ Community clinic/day hospital (primary care)<br>◯ Other
If OTHER, please specify the type of hospital or clinical setting you work in.	__________________

Do you treat children with epilepsy in your practice?	◯ Yes ◯ No
Do you report paediatric EEGs in your practice?	◯ Yes ◯ No
Have you had any previous paediatric EEG training?	◯ Yes ◯ No
If YES, during what time of your training (mark all that apply):-	☐ Registrar/residency rotation<br>☐ Fellowship program<br>☐ Online course<br>☐ Other
If OTHER, please specify in which settings EEG training was done?	______________________________
How long was the longest training period for?	◯ < 3 months<br>◯ 3-6 months<br>◯ 7-12 months<br>◯ Other
If OTHER, please specify how long was training for.	______________________________
What qualification(s) did you obtain? Mark all that apply:-	☐ Fellowship in EEGs<br>☐ Diploma in EEGs<br>☐ Online course certificates<br>☐ Other
If OTHER, please specify if any qualification was obtained.	______________________________

Current online handbook information

Question	Response
Have you completed the full online course?	◯ Partially ◯ Yes ◯ No
If NO, what was the primary reason why you did not finish the handbook?	◯ Handbook was too difficult<br>◯ Did not have time to complete<br>◯ Lost interest in handbook<br>◯ Subject difficult to learn<br>◯ Other
If OTHER, please specify why you did not finish the handbook.	__________________________________
If YES, please give the complete date of completion	__________________________________<br>(Please fill in month and year)
How long did it take you to complete the handbook from registration?	◯ < week<br>◯ 1-2 weeks<br>◯ 3-4 weeks<br>◯ Other
If OTHER, please specify how long it took to complete the handbook.	__________________________________
What was the primary reason for delay if completion took longer than one month?	◯ Busy schedule<br>◯ New subject<br>◯ Not user- friendly<br>◯ Internet / accessibility<br>◯ Other
If OTHER, please specify what was the reason for the delay in completing the handbook after a month.	__________________________________
Did you use the online facilities available for the book?	◯ Yes<br>◯ No
If YES, how useful was the chat?	__________________________________
If No, why did you not use the chat? Mark all that apply:-	☐ Did not need it<br>☐ Did not understand how to use it<br>☐ Had no access to internet<br>☐ Was not aware of availability of chat

Please complete the following section regarding your experience and opinion of the handbook. Please note that this is a Likert scale ranging from 1 through 5, with an option of 6 if unsure or not applicable. The higher the number the more positive the experience/opinion.

	Strongly Disagree	Disagree	Neutral	Agree	Strongly Agree	Unsure or not applicable
Before using the handbook, I was skilled in reading paediatric EEGs.	○	○	○	○	○	○

	Strongly Disagree	Disagree	Neutral	Agree	Strongly Agree	Unsure or not applicable
Using the handbook has improved my paediatric EEG reading skills overall	○	○	○	○	○	○

Please explain the reason(s) for the above rating:

____

	Strongly disagree	Disagree	Neutral	Agree	Strongly agree	Unsure or not applicable
Using the handbook has improved my paediatric EEG reading skills in: Identification of normal paediatric waveforms	○	○	○	○	○	○
Using the handbook has improved my paediatric EEG reading skills in: Identification of artifacts	○	○	○	○	○	○
Using the handbook has improved my paediatric EEG reading skills in: Identification of abnormalities(focal/generalized)	○	○	○	○	○	○
Using the handbook has improved my paediatric EEG reading skills in: Identification of hyperventilation/intermittent photic stimulation	○	○	○	○	○	○

Please explain the reason(s) for the above ratings:

____

	Strongly Disagree	Disagree	Neutral	Agree	Strongly Agree	Unsure or not applicable
The handbook is relevant to my current work.	○	○	○	○	○	○

	Strongly Disagree	Disagree	Neutral	Agree	Strongly Agree	Unsure or not applicable

I am applying what I learnt from the handbook to my current work.	◯	◯	◯	◯	◯	◯

Please explain the reason(s) for the above rating: ______________________

	Strongly Disagree	Disagree	Neutral	Agree	Strongly Agree	Unsure or not applicable
Using the handbook has improved the clinical care I am able to provide for my patients	◯	◯	◯	◯	◯	◯

Please explain the reason(s) for the above rating: ______________________

	Strongly Disagree	Disagree	Neutral	Agree	Strongly Agree	Unsure or not applicable
I would recommend this handbook and training program to others in the field	◯	◯	◯	◯	◯	◯

Please explain the reason(s) for the above rating: ______________________

Please list or describe any suggestions for improvement of this course OVERALL: ______________________

Please list and describe any suggestions for improvement of this course SPECIFICALLY related to ONLINE interface: ______________________

Please list and describe any positive aspects of taking this course: ______________________
