# Supplementary material for: Clinical practice applicability and relevance to non-specialists of a paediatric EEG online learning tool
Source: BMC Med Educ. 2024 Jan 31;24:102. doi: 10.1186/s12909-023-05017-2 (PMC10829391; doi:10.1186/s12909-023-05017-2)
Supplement: Supplementary file 2 — Additional file 2: Supplementary Figure 1. Countries with colour coding for number of non-responders (red 1–2; orange 3–4; and dark green 11–12). [file 12909_2023_5017_MOESM2_ESM.docx]

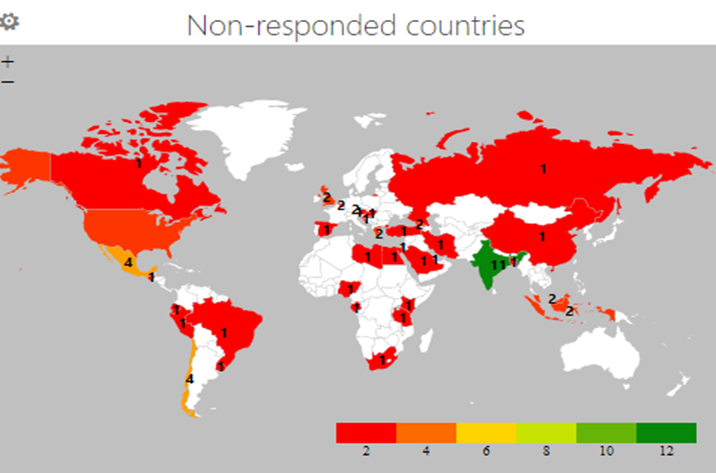


Supplementary figure 1: Countries with colour coding for number of non-responders (red 1-2; orange 3-4;

and dark green 11-12)
