# Supplementary material for: Clinical practice applicability and relevance to non-specialists of a paediatric EEG online learning tool
Source: BMC Med Educ. 2024 Jan 31;24:102. doi: 10.1186/s12909-023-05017-2 (PMC10829391; doi:10.1186/s12909-023-05017-2)
Supplement: Supplementary file 3 — Additional file 3: Supplementary Figure 2. Completion of online course by the country income grouping of the survey participants. [file 12909_2023_5017_MOESM3_ESM.docx]

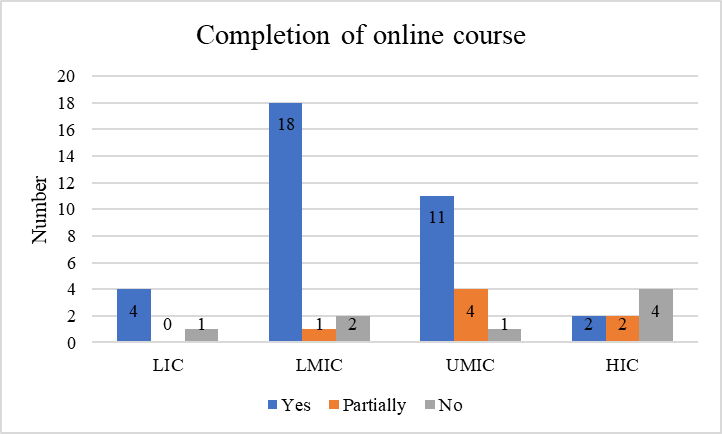


Supplementary figure 2: Completion of online course by the country income grouping of the survey participants
