# Supplementary figures and images for: Clinical practice applicability and relevance to non-specialists of a paediatric EEG online learning tool
Source: BMC Med Educ. 2024 Jan 31;24:102. doi: 10.1186/s12909-023-05017-2 (PMC10829391; doi:10.1186/s12909-023-05017-2)

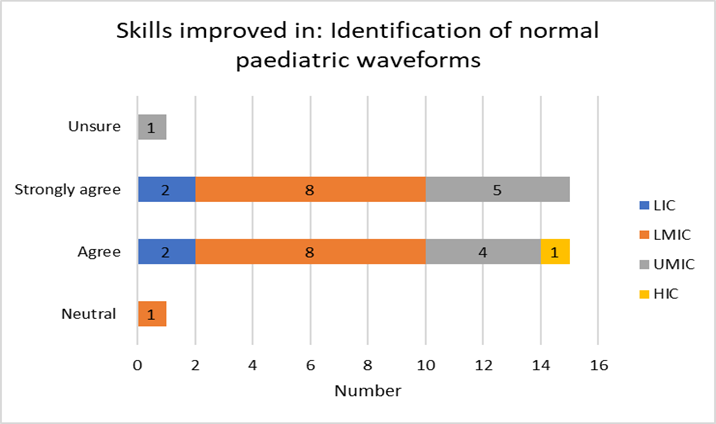


Supplementary figure 3.1: Identification of normal waveforms n= 32

Supplement: Supplementary file 4 — Additional file 4: Supplementary Figure 3.1. Identification of normal waveforms n = 32. Supplementary Figure 3.2. Identification of artifacts n = 32. Supplementary Figure 3.3. Identification of abnormalities n =32. Supplementary Figure 3.4. Identification of activation procedures n = 32. [file 12909_2023_5017_MOESM4_ESM.zip › Supplementary figure 3.1.docx]

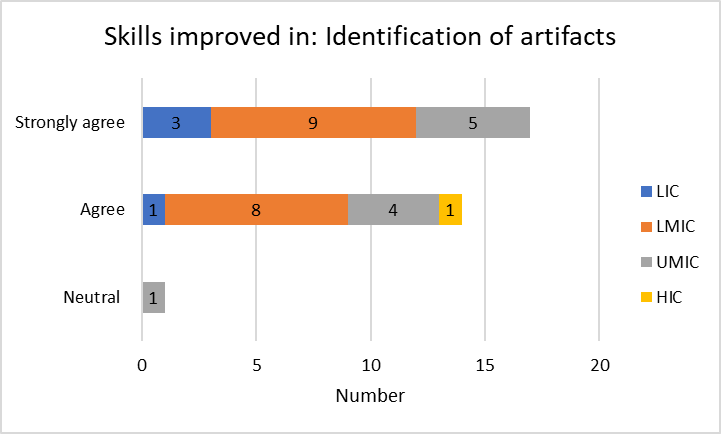


Supplementary figure 3.2: Identification of artifacts n= 32

Supplement: Supplementary file 4 — Additional file 4: Supplementary Figure 3.1. Identification of normal waveforms n = 32. Supplementary Figure 3.2. Identification of artifacts n = 32. Supplementary Figure 3.3. Identification of abnormalities n =32. Supplementary Figure 3.4. Identification of activation procedures n = 32. [file 12909_2023_5017_MOESM4_ESM.zip › Supplementary figure 3.2.docx]

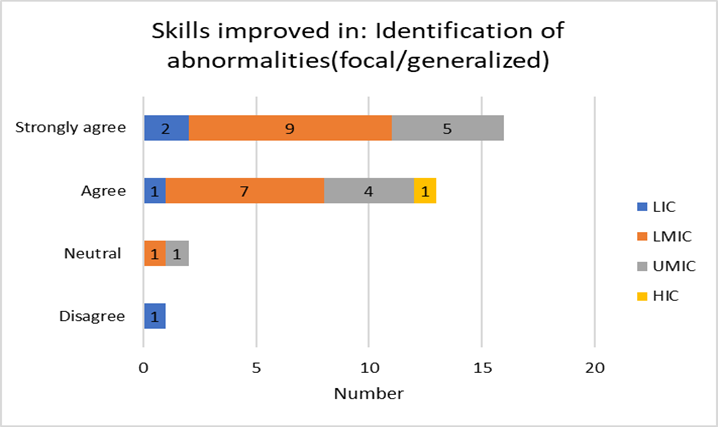


Supplementary figure 3.3: Identification of abnormalities n=32

Supplement: Supplementary file 4 — Additional file 4: Supplementary Figure 3.1. Identification of normal waveforms n = 32. Supplementary Figure 3.2. Identification of artifacts n = 32. Supplementary Figure 3.3. Identification of abnormalities n =32. Supplementary Figure 3.4. Identification of activation procedures n = 32. [file 12909_2023_5017_MOESM4_ESM.zip › Supplementary figure 3.3.docx]

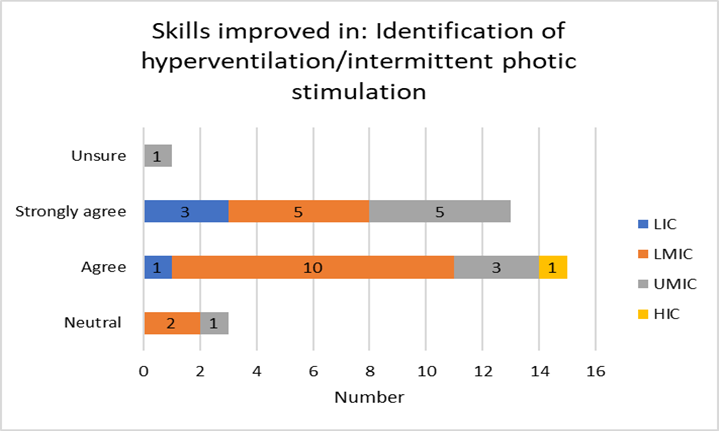


Supplementary figure 3.4: Identification of activation procedures n=32

Supplement: Supplementary file 4 — Additional file 4: Supplementary Figure 3.1. Identification of normal waveforms n = 32. Supplementary Figure 3.2. Identification of artifacts n = 32. Supplementary Figure 3.3. Identification of abnormalities n =32. Supplementary Figure 3.4. Identification of activation procedures n = 32. [file 12909_2023_5017_MOESM4_ESM.zip › Supplementary figure 3.4.docx]

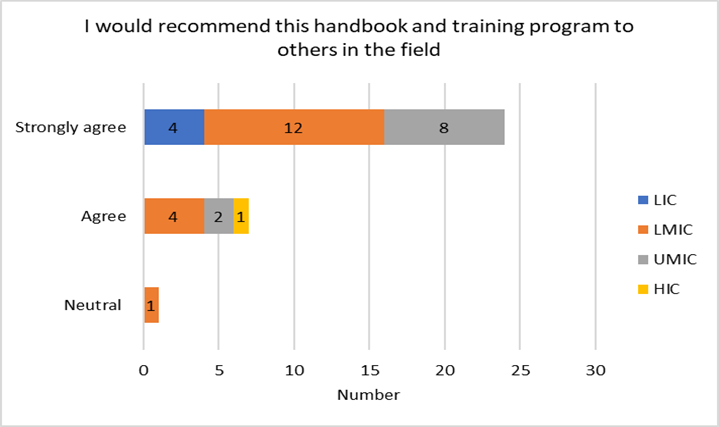


Supplementary figure 4: Recommendation of handbook and training program n=32

Supplement: Supplementary file 5 — Additional file 5: Supplementary Figure 4. Recommendation of handbook and training program n = 32. [file 12909_2023_5017_MOESM5_ESM.docx]
